# Genomic sequence identification of *Arthrobacter* phage Ascela

**DOI:** 10.1128/MRA.00776-23

**Published:** 2023-10-31

**Authors:** Audrey E. Nesbit, Alison E. Kanak

**Affiliations:** 1 Department of Biology, University of North Georgia, Dahlonega, Georgia, USA; Portland State University, Portland, Oregon, USA

**Keywords:** bacteriophages, bacteriophage genetics, *Arthrobacter*

## Abstract

*Arthrobacter* phage Ascela was isolated in North Georgia. Its genome is 44,192 bp with 71 open reading frames and a GC content of 67.4%. It shares 99.29% nucleotide identity with *Arthrobacter* phage Iter. Actinobacteriophages that share over 50% nucleotide identity are sorted into clusters, with Ascela in cluster AZ and subcluster AZ1.

## ANNOUNCEMENT

There is a growing concern about antibiotic-resistant bacteria, which could be addressed by phage therapy. The discovery and characterization of bacteriophage are important because they could be used for treatments of bacterial infections. Here, we present the *Arthrobacter* phage Ascela, isolated using a Science Education Alliance–Phage Hunters Advancing Genomics and Evolutionary Science protocol (1).

**Fig 1:**
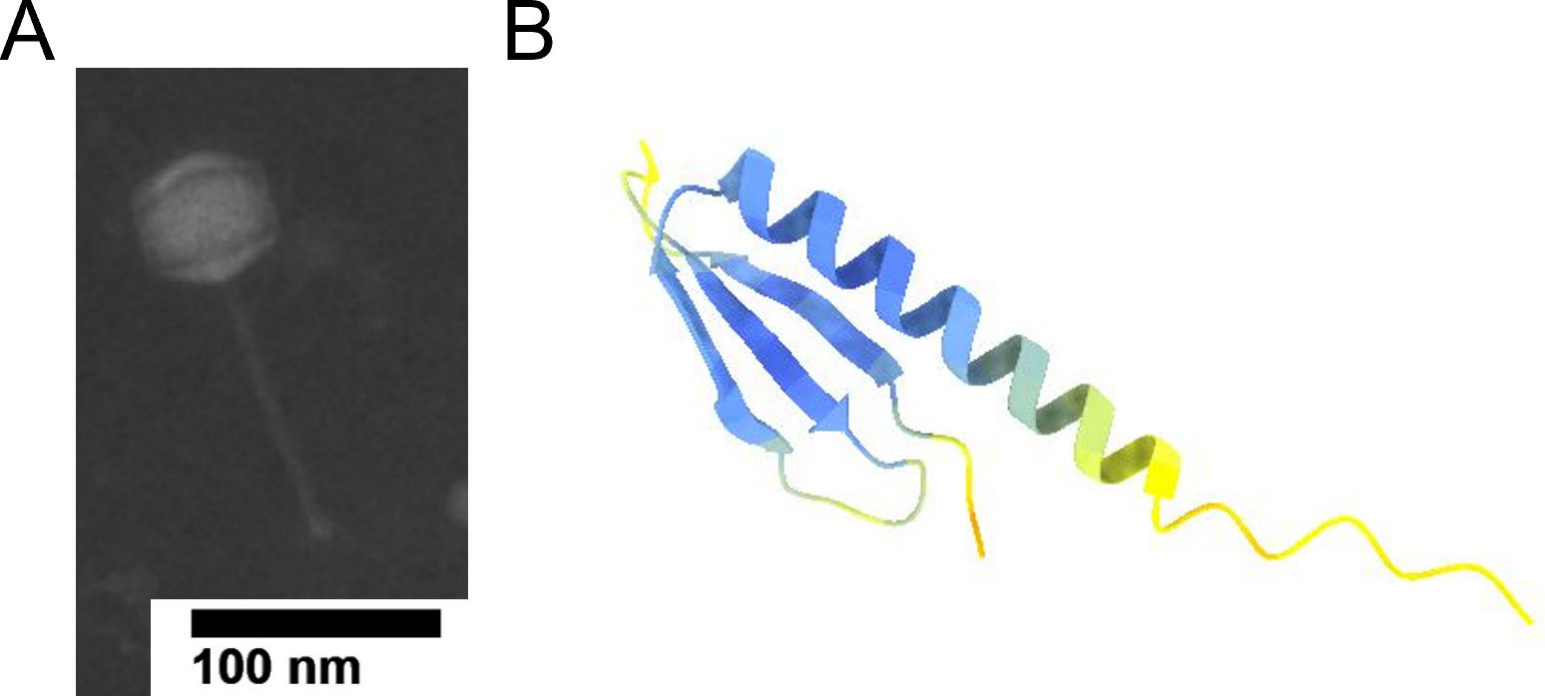
**(A)** Transmission electron micrograph (TEM) of Ascela. TEM images were obtained using a JEM-1011 TEM (JOEL, Inc., Tokyo, Japan) at the University of Georgia Electron Microscope laboratory. (B) Structure prediction of Ascela ORF59. Potential protein tertiary structure was predicted using AlphaFold (5).

Ascela was isolated from shallow, lake-side soil collected in Dahlonega, Georgia (34.551799˚N, 83.966918˚W) in 2021. *Arthrobacter globiformis* B-2979 was used for Ascela’s isolation from the environmental sample. Briefly, LB liquid medium was added to soil, incubated at 30°C for 24 hours, and filtered using a 0.22-µm filter. Phage presence was confirmed and purified via standard plaque assay. After three rounds of purification, phages were amplified to a high titer to extract phage genomic DNA for sequencing (2). Electron microscopy using phosphotungstic acid as a negative stain identified Ascela as having siphovirus morphology with a capsid and tail measuring 45.6 nm in diameter and 113.5 nm in length, respectively (Fig. 1A). Ascela’s plaques are regularly 3 mm in diameter with distinct margins, but plaques with other characteristics were also observed. Actinobacteriophages that share over 50% nucleotide identity are sorted into clusters and subclusters where appropriate (3, 4). Ascela was sorted into cluster AZ and further into subcluster AZ1.

Phage genomic DNA was extracted from Ascela lysate with a Wizard DNA extraction kit (Promega) per the manufacturer’s instructions. An NEB Ultra II Library Kit with v3 Reagents and 150-base single-end reads was used to assemble a sequencing library. Ascela was run using Illumina MiSeq sequencing. Ascela’s coverage was 249× with no Sanger finishing reactions required. These raw reads were assembled using Newbler v2.9 (Rosche) and Consed version June 2022 (6). The resulting single phage contig was checked for completeness, accuracy, and phage genomic termini using Consed v29 as previously described.

The genome was annotated using GeneMark v3.25 (7), NCBI BLAST v2.13.0, Glimmer v3.02, HHpred v3.2.0 (8, 9), ARAGORN v1.2.38 (10, 11), and Phamerator (5). Default parameters were used for all software. Hits with E values of 10^e-10^ or less were considered acceptable. Phamerator and GeneMark indicate that Ascela has 71 open reading frames (ORFs), and functions were able to be predicted for 34 of them. All genes are transcribed in the forward direction except for genes 38 and 50, which are transcribed in the reverse direction. Ascela is predicted to be a temperate phage, as a predicted serine integrase (ORF51) was identified. A phamily was determined using Phamerator by using “pairwise comparisons to generate gene relationships” (5). Ascela has 3′ sticky ends with an 11-bp overhang. Ascela is most genetically similar to Iter (GenBank accession no. ON208833), having 99.29% nucleotide identity via BLAST alignment. There is a single gene insertion in Ascela’s genome at ORF59 with no known function able to be called. The structure predicted for ORF59 has a single alpha helix 24 amino acids long at the C terminus and a single beta sheet at the N terminus as predicted by Alphafold (12, 13) (Fig. 1B).

## Data Availability

Information on Ascela’s genome can be found in GenBank under accession no. OQ709218. Sequencing reads are part of the Sequence Read Archive with accession no. SRX20165771 under BioProject accession no. PRJNA488469.
